# Estimating Water Transport from Short-Term Vessel-Based and Long-Term Bottom-Mounted Acoustic Doppler Current Profiler Measurements in an Arctic Lagoon Connected to the Beaufort Sea

**DOI:** 10.3390/s22010068

**Published:** 2021-12-23

**Authors:** Chunyan Li, Kevin Mershon Boswell

**Affiliations:** 1 Department of Oceanography and Coastal Sciences, College of the Coast and Environment, Louisiana State University, Baton Rouge, LA 70803, USA; 2 Institute of Environment, Florida International University, North Miami, FL 33181, USA; kevin.boswell@fiu.edu

**Keywords:** acoustic Doppler meters, ocean current, transport to the Arctic, moving platform measurements, regression with fixed sensors

## Abstract

Acoustic Doppler current profilers (ADCP) are quasi-remote sensing instruments widely used in oceanography to measure velocity profiles continuously. One of the applications is the quantification of land–ocean exchange, which plays a key role in the global cycling of water, heat, and materials. This exchange mostly occurs through estuaries, lagoons, and bays. Studies on the subject thus require that observations of total volume or mass transport can be achieved. Alternatively, numerical modeling is needed for the computation of transport, which, however, also requires that the model is validated properly. Since flows across an estuary, lagoon, or bay are usually non-uniform and point measurements will not be sufficient, continuous measurements across a transect are desired but cannot be performed in the long run due to budget constraints. In this paper, we use a combination of short-term transect-based measurements from a vessel-mounted ADCP and relatively long-term point measurements from a moored ADCP at the bottom to obtain regression coefficients between the transport from the vessel-based observations and the depth-averaged velocity from the bottom-based observations. The method is applied to an Arctic lagoon by using an ADCP mounted on a buoyant platform towed by a small inflatable vessel and another ADCP mounted on a bottom deployed metal frame. The vessel-based measurements were performed continuously for nearly 5 h, which was sufficient to derive a linear regression between the datasets with an R^2^-value of 0.89. The regression coefficients were in turn applied to the entire time for the moored instrument measurements, which are used in the interpretation of the subtidal transport variations.

## 1. Introduction

Doppler shift is a physical phenomenon recognized about 180 years ago [1]. When a source of waves and a receiver of those waves have a relative motion, the received frequency is dependent not only on the original frequency sent out from the source but also on the relative velocity between the source and receiver [2]. This change in frequency due to the relative motion is the Doppler shift or Doppler effect. The most common Doppler shift phenomena include those for electromagnetic waves and acoustic waves [3].

Doppler shift has been applied in technology for instrumentation in many fields [4]. For example, Doppler radar for weather has been widely used for real-time monitoring of precipitation and severe weather, including thunderstorms and tornadoes [5,6,7,8,9]. The U.S. is now equipped with ~159 Next-Generation Radars (NEXRAD [10,11]) for weather service. 

The high frequency (HF) radar [12,13,14,15,16] is another example for ocean surface current measurements. There are about 150 HF radars along the coast of the U.S., including the Great Lakes, and the data are reported in real-time to the U.S. Integrated Ocean Observing System [17]. These systems provide continuous coverage of surface flows in regions of interest for data used for research and forecast purposes.

To measure the vector field of the flow, a single wave source (the transducer) is not enough. A Doppler radar with a single wave source can only measure the speed in the radial direction but not the velocity vector because it cannot measure the velocity component in the crossbeam direction. By using two or more wave sources (transducers), a velocity vector field can be resolved as the crossbeam velocity component can be accounted for by the other transducer(s). This is achieved in the weather radar [18] and HF Radar [13] applications by overlapping the area of coverage of two adjacent radar units.

Doppler shift has also been used in acoustic waves for flow velocity measurements in the ocean. Started in the 1980s [19], commercial acoustic Doppler current profilers (ADCPs) have been applied to measure vertical profiles of 3-D velocity vectors (with east, north, and vertical components) using three to nine transducers (or beams) integrated into an underwater enclosure. The multi-beam transducers are designed to have slightly different angles allowing a mathematical solution (a matrix inversion) to compute the velocity vector. More transducers (>3) allow extra degrees of freedom for more information such as error estimates as well as for increased accuracy.

ADCPs have been widely used in measurements of ocean current velocity or suspended sediment profiles either along the vertical [20] or along the horizontal [21,22]. They can be mounted on fixed platforms, deployed on moorings on the bottom or used on moving platforms [23,24,25,26] (ships or other automated, tethered, or remotely controlled, or programmed survey vehicles). One of the applications of ADCPs is the measurements of water transport across a waterway between different regions in the ocean or an estuary [23,24,25]. The cross-sectionally integrated transport of water, salt, suspended sediments, nutrients, and other bio-geo-chemical materials is of great importance in studying the land-ocean exchange, the effect of climate change, and anthropogenic impact on the coastal and global environment.

Global climate change has shown a greater magnitude of trend of warming in the Arctic region [27,28,29]. As a result, more freshwater is transported from the permanent ice in the ocean and on land to the Beaufort Sea [30] and other regions in the Arctic Ocean. The study of this requires a combined effort using satellite remote sensing, numerical experiments, and in situ observations of water transport between the Arctic land and Arctic Ocean through rivers, estuaries, and lagoons, which are abundant in the region and yet have been the least measured due to the logistical challenges of surveying in the coastal Arctic.

**Figure 1:**
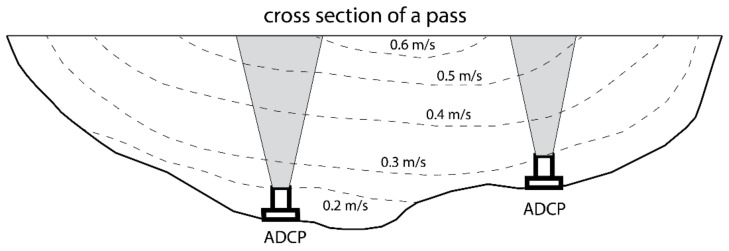
Using bottom-mounted ADCP(s) to measure flow profiles and estimate cross-sectional transport. Contour lines are hypothetical flow velocity magnitudes across the section (flow is into or out of the plane).

The present study is motivated by this larger and longer scope of research, using ADCPs to quantify the cross-sectional water transport through an Arctic lagoon. The work alone is not able to reach the ultimate goal of the evaluation of the impact of climate change and Arctic warming. Rather, it is aimed at the development of an effective method for conducting reliable measurements using acoustic sensors. One of the challenges of the transport measurement is that the spatial coverage and temporal coverage cannot be satisfied at the same time. As shown in Figure 1, a common practice of long-term measurements of transport of water across a waterway is to use bottom-mounted ADCP(s). Because the velocity field is often non-uniform, e.g., with greater velocity in the center of the channel and smaller velocity over the shallow shoals [23,24,25,31,32], it is impossible to have reliable cross-sectional transport represented by 1 or 2 bottom-mounted ADCP(s). At most, they provide a proxy, but the error is difficult to obtain without additional information.

This leads to the design of observations during which measurements are performed simultaneously from a vessel-based ADCP transecting along a cross sectional line and a bottom-mounted ADCP deployed at a location representative of the flow inside the channel. A statistical relationship is then established between the two datasets such that the bottom-mounted ADCP data can be used to rescale to the total transport for a longer time period. This paper will introduce such a method applied to a tidal inlet of an Arctic lagoon (Elson Lagoon). The next section discusses the details of the method, the implementation of the measurements, followed by Section 3 on the major results, Section 4 with discussion, and Section 5 for the conclusions.

## 2. Materials and Methods

### 2.1. Study Site

**Figure 2:**
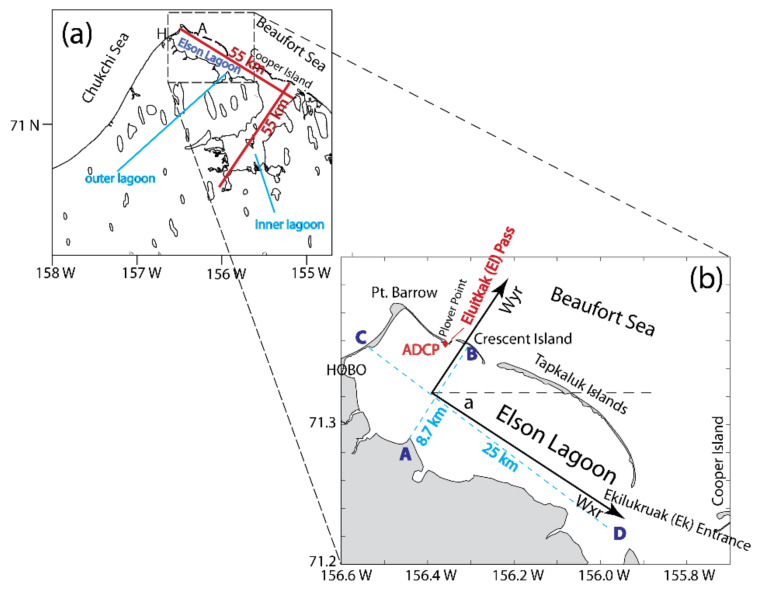
Study area at the northwestern Alaska between the Chukchi Sea and Beaufort Sea (**a**). The zoomed-in view of the Elson Lagoon (**b**) which also shows the scales and the location of bottom-mounted ADCP at the Plover Point at Eluitkak Pass.

Our experiment was conducted in Elson Lagoon in northern Alaska. It is the northernmost coastal lagoon-estuary of the United States. The northwestern portion of this system is a rectangle of ~8 × 25 km and the mean water depth is about 2–3 m (Figure 2). The lagoon is located near the confluence of the Chukchi and Beaufort Seas. It is roughly bounded within 156°36′ W, 155°54′ W, 71°12′ N, and 71°23′ N and oriented in the northwest–southeast direction. Eluitkak Pass, in the northwestern corner of the lagoon, is a relatively wide (~300 m) and deep (~16 m) channel and is where we deployed the bottom-mounted ADCP and performed the vessel-based transects. A chain of islands located east and southeast of the Eluitkak Pass is along the coast as the seaward boundary of the lagoon connecting to the coastal ocean and the Beaufort Sea. This is a region strongly influenced by the Arctic lows and highs of air pressure systems and severe storms [33].

### 2.2. Instruments

**Figure 3:**
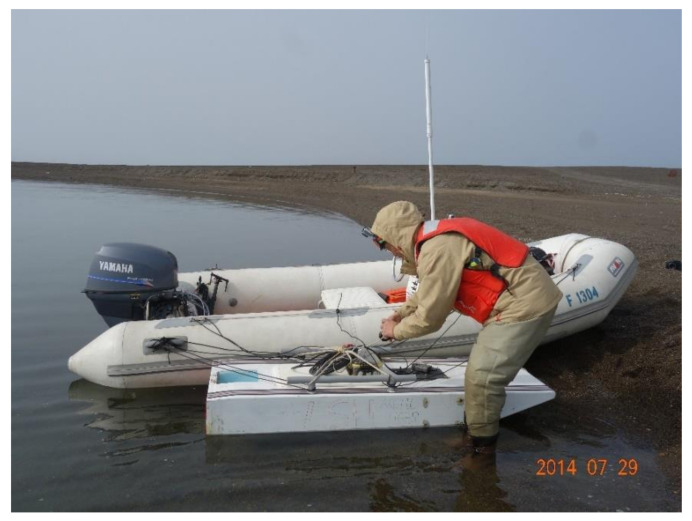
The inflatable vessel used to tow the fiberglass surface craft (in front of the inflatable vessel), which carried the M9 ADCP.

In this study, we used two ADCPs. One of them was deployed on the bottom of the Eluitkak Pass. The second one was mounted on a fiberglass surface craft. This surface craft carrying the ADCP was towed on the starboard side of an inflatable boat (Figure 3) measuring the flow velocity profiles and the cross-channel total transport at Eluitkak Pass.

The bottom-mounted instrument was a 1200 kHz Teledyne RD Instrument Workhorse ADCP, which has four beams with a Janus configuration. The vessel-mounted system was a Sontek multi-frequency M9 ADCP configured with 9-beams, four of them working at 2 MHz frequency while four other beams working at 1 MHz. The last beam was the vertical one working at 0.5 MHz to measure the water depth. To record the position of the boat, a Garmin GPSmap 60CSx was used with differential GPS.

### 2.3. Measurements

The mooring was comprised of an aluminum cross with 5 pounds of extra weight on each of the four “legs” for stability, on which the ADCP was mounted in an upward direction. The ADCP’s compass was calibrated before the deployment according to the manufacturer’s procedures. The ADCP’s internal clock was set to record in UTC. It was deployed at (71.3593° N, 156.3561° W, on the western side of the inlet) at a depth of about 13.35 m, on 29 July 2014. With a blanking distance of ~1 m, the bottom most data point was at 1.53 m and the vertical interval of measurement was 1.00 m. The ADCP was setup to sample once every 80 s and 45 times per hour (Table 1). The averaged hourly data were saved in the internal memory. The starting time of valid data was 1630 UTC 29 July 2014 and the last valid ensemble of measurements was at 0230 UTC, 3 August 2014 for the first deployment. The second deployment of this ADCP was made about one day later with the valid data between 0320 UTC 4 August 2014, and 1800 UTC, 13 August 2014. The instrument was deployed at a slightly different location (71.3597° N, 156.3538° W), northeast of the first location at a depth of 11.00 m. The second deployment also had a different setup for the sampling schemes. Specifically, the vertical intervals were changed to 0.25 m (instead of 1.00 m) and the ensemble sampling time interval was set to 5 min, within which 50 samples would be taken at 6 s intervals for the 5 min ensemble average (Table 1).

The M9 ADCP’s compass was calibrated according to the manufacturer’s suggested procedures. The internal clock of the M9 was set to record in UTC. About seven hours after the bottom-mounted ADCP was deployed, just prior to 1630 UTC on 29 July 2014, the inflatable vessel towing the M9 ADCP started to run across the ~300 m wide Eluitkak Pass. For the entire measurement period, the M9 ADCP was operating under the “bottom tracking mode”, which used the strong bottom return signal originally sent from the ADCP transducers to compute the instantaneous velocity of the vessel relative to the sea bottom. This bottom tracking velocity was then used to compute the Earth-coordinate velocity components for the water particles. This approach gives a higher accuracy for the velocity field of the water, compared to using the raw GPS data for the same purpose: if the real-time raw GPS data were used to compute the velocity of the vessel, it would have introduced a much greater error in the water velocity computation.

Data collection by the M9 ADCP commenced at about 2322 UTC. This was performed concurrently with the Garmin GPS recording data at 1 s intervals. The inflatable vessel was running across the Eluitkak Pass repeatedly for 41 times until 0414 UTC 30 July 2014. During this time period, the average speed of the boat was estimated at about 0.7 m/s. The transect across Eluitkak Pass passed the bottom-mounted ADCP so the flow velocities from both boat-based and bottom deployed ADCPs can be directly compared.

Note that it was a challenge and risky operation to run an inflatable boat in that remote area. The fog, winds, waves, and cold air and water make it difficult to maintain the transect lines in the small inflatable vessel; however, in consideration of these conditions, the survey and data collection were quite successful.

### 2.4. Data Processing

The M9 ADCP data included accurate time stamps but not the geo-location. With the time series of vessel positions from the Garmin GPS, we merged the GPS data with the ADCP data by simple interpolations. Since both M9 and Garmin GPS recorded data at 1-s intervals, they were comparable in time increments, and the interpolation maintained the quality of the data.

**Figure 4:**
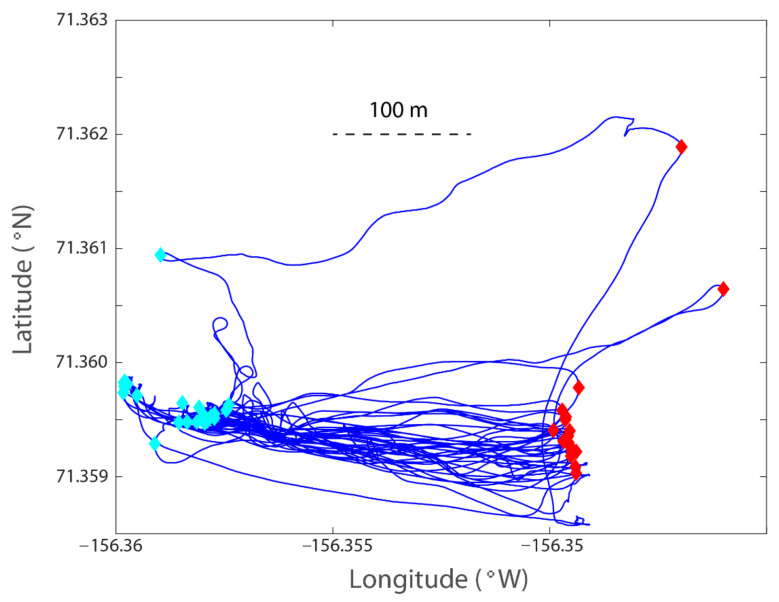
Vessel track and points selected for the west and east ends of a transect for total cross-sectionally integrated transport. The light blue and red diamonds represent the west and east end of the transects, respectively. Four of the forty-one lines were excluded in the analysis as they were too far away from the intended transect. A horizontal scale of 100 m is indicated.

**Figure 5:**
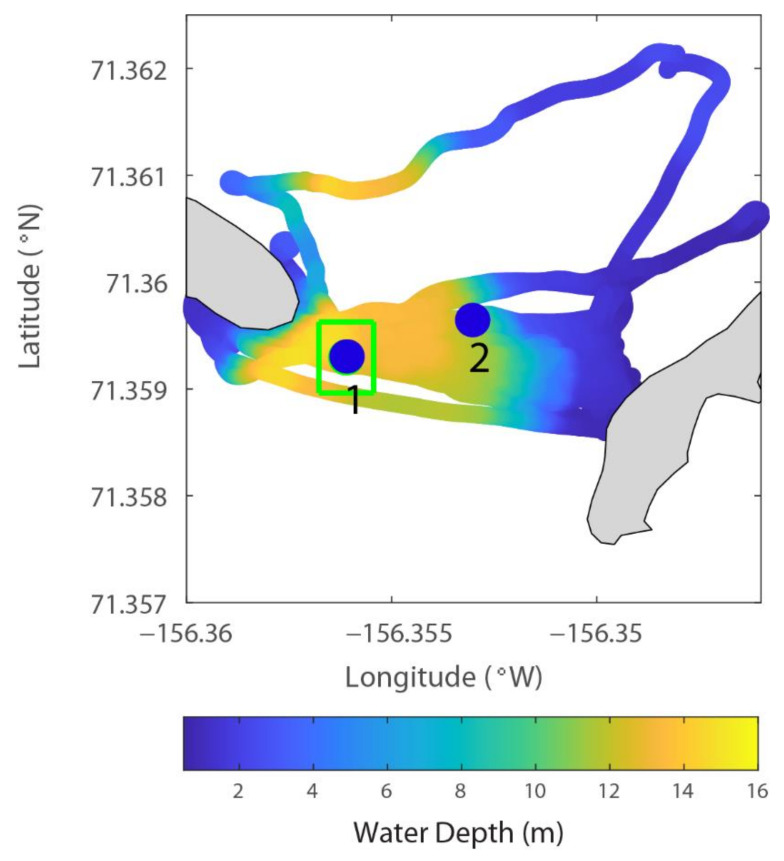
ADCP locations and water depth measured by the vessel-based M9 ADCP. The locations of the bottom-mounted ADCP are shown by the blue circles and the numbers (1 and 2) indicate the first and second deployments, respectively. The green rectangular box shows the region selected for capturing the flow velocity data from the vessel-based ADCP to compare with the flow data from the first mooring.

The M9 ADCP data provide outputs for both velocity profiles and integrated total transport across the channel. To extract the total transport, the start and end points of each transect must be determined. This was completed for all the 41 repetitions across the channel (Figure 4). Among the 41 transects, we selected 37, excluding 4 that were too far away from the intended transect. The water depth measured from the vessel-based ADCP is shown in Figure 5. The main channel is on the western end, and the eastern end has a relatively wider shoal of 2–5 m.

To extract the velocity data from the vessel-based ADCP to compare with those from the bottom-mounted ADCP, we defined a rectangle around the deployed ADCP (Figure 5, green box) with a dimension of roughly 74 m in the north–south direction and 47 m in the east–west direction. This 74 × 47 m data footprint (the green box) is a proper selection as the water depth within this box is consistent (varying within 2 m) and the vessel’s speed (averaged ~0.7 m/s) is slow enough to permit sufficient sampling inside the box for a reliable statistical averaged velocity each time the vessel passed through the box.

The middle (average) time of each of the transects was used to interpolate the bottom ADCP data onto the same time of the vessel-based transport measurements (37 transport values). This was performed to create a time series for the velocity vector at each of the vertical locations, as well as the depth-averaged velocity. This permits the development of a regression between the velocity from the bottom-mounted ADCP and the boat-based transport measurements:

(1) $$P=\alpha_{1}v+\alpha_{2}$$

in which P is the cross-channel volume transport measured from the vessel, v is the north component of the velocity measured by the bottom-mounted ADCP, and $\alpha_{1}$ and $\alpha_{2}$ are the regression coefficients. Note that different sets of $\alpha_{1}$ and $\alpha_{2}$ are expected for velocities from different vertical positions and for the depth-averaged velocity. The east component of the velocity from the bottom ADCP was not used as it was roughly in the cross-channel direction and not correlated with the transport.

If the correlation between the two components is high, we can use the regression coefficients to compute the transport from the velocity time series measured by the bottom-mounted ADCP. This will be useful because the bottom-mounted ADCP was deployed for more than one week while the vessel-based survey would not be safe to run at night, and it is impossible to continue for several days, considering the challenging Arctic conditions there.

Since the second deployment was at a slightly shallower depth (~11.00 m) than the first (13.35 m), the velocity magnitude measured is affected by the depth difference. As demonstrated in [34] for the mean flow or low frequency (quasi-steady-state) flows and [32] for tidal flow, the velocity magnitude is dependent on water depth. When compared along *the same cross section*, assuming all other parameters are the same, the shallower water will have a smaller velocity magnitude for both low frequency (or mean) flow and tidal flow. Thus, we need to find the velocity factors to transform the velocity at the second site of deployment to that at the first site so the regression coefficients can be applied to compute the total cross-sectional transport for the entire time periods of both deployments. More specifically, for the low-frequency flow component [34],

(2) $$u_{1}=u_{2}\sqrt{\frac{h_{1}}{h_{2}}}$$

in which $u_{1}$ and $u_{2}$ are the velocities at site 1 and 2, with depth $h_{1}$ and $h_{2}$, respectively. In our case, $h_{1}=13.35$ m, $h_{2}=11.00$ m, so the factor for low-frequency velocity transformation from site 2 to site 1 is

(3) $$f_{1}=\sqrt{\frac{h_{1}}{h_{2}}}=1.1017$$

For the tidal flow, from [32], the factor for velocity transformation from site 2 to site 1 is

(4) $$f_{2}=\frac{h_{1}}{h_{2}}\sqrt{\frac{\sigma^{2}h_{1}^{2}+\beta^{2}}{\sigma^{2}h_{2}^{2}+\beta^{2}}}$$

in which σ is the angular frequency for tide, which in this region is semi-diurnal, or

(5) $$\sigma =\frac{2\pi }{12\times 3600}\;\;\;\left(\mathrm{rad}/s\right)$$

and β is a friction coefficient defined by [35,36]

(6) $$\beta =\frac{8C_{D}U_{0}}{3\pi }$$

where $C_{D}$ and U are the bottom drag coefficient and tidal velocity amplitude, respectively. In this study, we choose the typical value for the drag coefficient $C_{D}=0.0025$ [36], and $U_{0}=0.5$ m/s based on our data. This yields,

(7) $$f_{2}=1.053$$

Therefore, to transform the velocity measured from site 2 to site 1, the low-frequency component should be increased by about 10% ($f_{1}=1.1017$) and the tidal component by about 5% ($f_{2}=1.053$).

To transform the velocity measured at site 2 during the second deployment to that at site 1 so we can implement the regression coefficient for the transport computation for the second deployment period, we low-pass filtered the data from the second deployment to separate the tidal and non-tidal (low frequency) velocity components. For that purpose, a 40-h Butterworth [37] low-pass filter was used for the velocity data from the second deployment to separate the time series of depth-averaged velocity $v_{2}$ into

(8) $$v_{2}=v_{L}+v_{T}$$

Here, $v_{L}$ and $v_{T}$ are the low-frequency and tidal velocity components, respectively. The reason we used a 40-h cut-off for the low-pass filter is because we need to filter out diurnal tidal constituents. Although tide in the region is basically semi-diurnal, there is diurnal inequality and thus diurnal constituents are not exactly zero. Using a 40-h cut-off can eliminate any diurnal tide and eliminate any sidelobe leakage effect.

By applying the factor $f_{1}$ to $v_{L}$ and $f_{2}$ to $v_{T}$, an approximated velocity at site 1 for the second period can be obtained before applying the regression coefficients to obtain the total transport:

(9) $$v_{1}=f_{1}v_{L}+f_{2}v_{T}$$

The total transport is then

(10) $$P=\left(v_{1}\;I\right)\left(\begin{array}{c} \alpha_{1}\\ \alpha_{2} \end{array}\right)$$

Here, I is an array of 1′s of N×1 dimension, in which N is the length of the time series $v_{1}$ or $v_{2}$ (same length), and $\alpha_{1}$ and $\alpha_{2}$ are the regression coefficients in (1).

## 3. Results

### 3.1. Velocity Comparison

**Figure 6:**
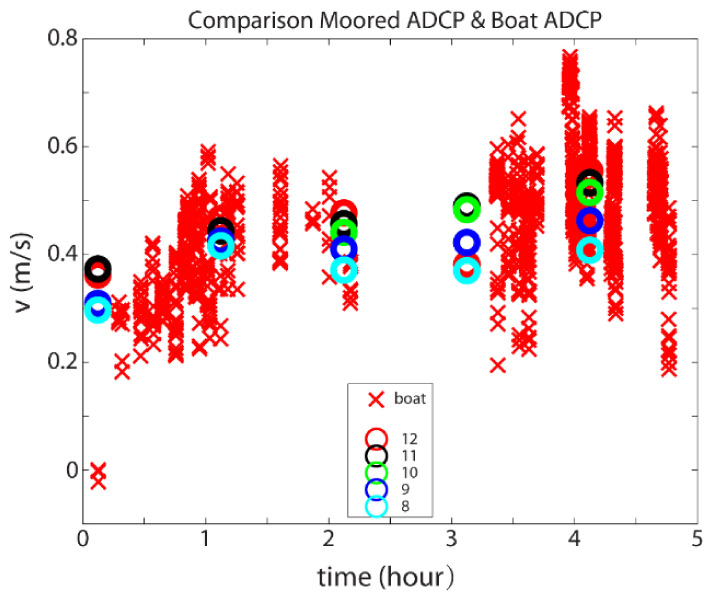
Comparison between velocities. Red crosses are the north velocity component from the vessel-based ADCP at different depths. The colored circles are the velocity data from the bottom-mounted ADCP at different heights above the bottom (e.g., the red circle is 12 m above the bottom, black circle is 11 m above the bottom, etc.).

The velocity measured from the bottom-mounted ADCP during the transect measurement varied between 0.3 and 0.55 m/s. The vessel-based ADCP measured greater velocity variance. This was expected because the transects covered a much larger area, including the shallow waters on the eastern end and the deepest channel (~16 m), which was slightly northwest of the bottom deployed ADCP at site 1 (within 30–50 m range, Figure 5). Nevertheless, the velocity from these two ADCPs showed consistency (Figure 6).

### 3.2. Regression of Transport

**Figure 7:**
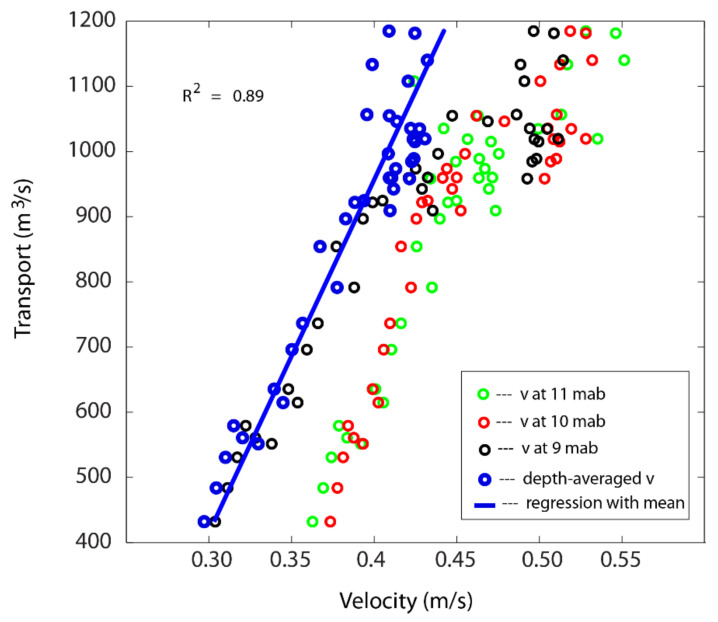
Linear regression between the velocity measured by the bottom-mounted Teledyne RDI ADCP at site 1 with the total cross channel transport measured by the vessel-based M9 ADCP during the first deployment. The circles are for velocity from the bottom-mounted ADCP at various heights (meter above the bottom, or mab). The blue circles are the depth-averaged velocity from the bottom-mounted ADCP.

The horizontal velocity showed a dependency on vertical position: the nearer the bottom, the lower the velocity. As the depth-averaged velocity varied from 0.3 to 0.45 m/s, the transport tripled from 400 to 1200 m^3^/s. Compared with the regression with velocities at different vertical positions, the depth-averaged velocity had the best correlation with the total transport from the vessel-based measurements with an R^2^ value of 0.89 (Figure 7). The coefficients $\alpha_{1}$ and $\alpha_{2}$ were 4801.9 and −971.8, respectively. The 95% confidence intervals for these two parameters were (4214.6, 5389.3) and (−1200.7, −742.9), respectively. From this result, we can see that the correlation between the two instruments is significant. The negative value for $\alpha_{2}$ indicates that there is an inward transport when the depth-averaged velocity from the bottom-mounted ADCP site is 0. This may imply that there is an inward flow in the shallow waters away from the ADCP site when the flow in the channel is small.

### 3.3. Transport Time Series

**Figure 8:**
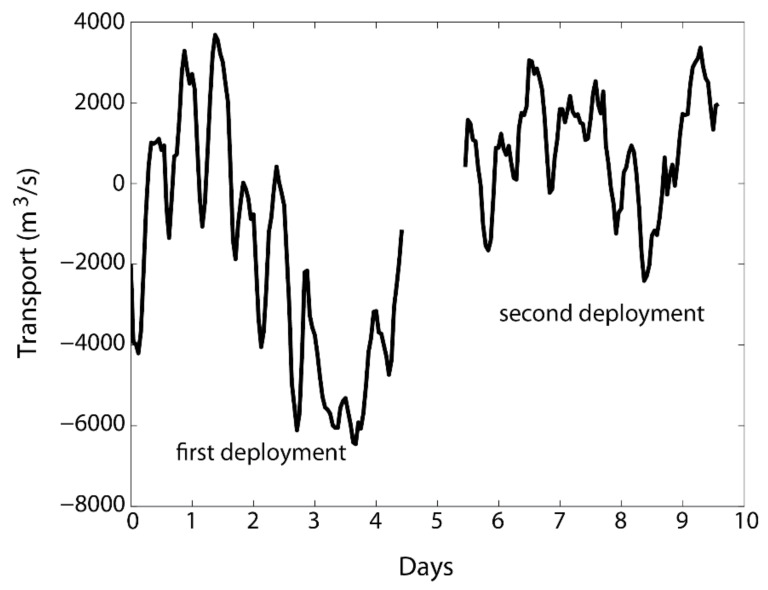
Transport computed for the first and second deployments. The horizontal axis is time in days from the beginning of the first deployment.

Using the regression coefficients $\alpha_{1}$ and $\alpha_{2}$, the transport for the entire time of the first deployment (about 4.5 days) can be calculated based on the depth-averaged velocity from the bottom-mounted ADCP at site 1. Applying Equations (8)–(10), the transport for the entire time of the second deployment (about 4 days) can also be calculated based on the depth-averaged velocity from the bottom-mounted ADCP at site 2. The maximum outward transport (positive sign means outward transport) was about 3800 m^3^/s during the first deployment (Figure 8). There was a strong outward flow between days 3 and 4 during the first deployment when the total transport was greater than 6000 m^3^/s into the lagoon (negative sign). This turns out to be a significant event because of a northwesterly wind associated with an Arctic low air pressure system (see below in the discussion section) that induced inward transport through the multiple inlet hydrodynamics [22,38] as well as Ekman transport. The second deployment period did not have such strong inward transport, but the outward transport was comparable. Furthermore, semi-diurnal tidal signals are obvious for both periods of deployment. The transport from this project showed that the non-tidal variation amounts to 10,000 m^3^/s (−6000 to 4000), while the tidal variations only had 30–50% of that, indicating that in this region, the wind-driven flows are more important than those of the tidal motions.

## 4. Discussion

### 4.1. Weather Conditions

**Figure 9:**
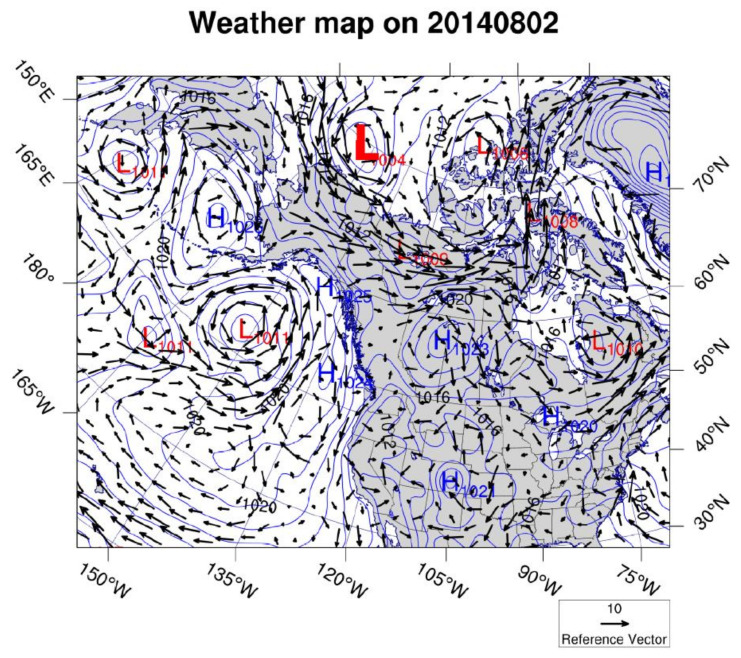
Weather map from NOAA’s reanalysis data for 2 August 2014. The low air pressure system indicated by a large red “L” in the upper portion of the map indicates the wind (to the southwest of the “L”) was from the northwest, suggesting Ekman transport into the Elson Lagoon.

Our study happened to coincide with a strong atmospheric low-pressure system passing north of the region in the Beaufort Sea (Figure 9) one day before the retrieval of the ADCP during the first deployment. A cyclonic wind associated with the low atmospheric system brought a strong northwesterly wind to the Elson Lagoon (Figure 9). This produced a significant inward transport exceeding 6000 m^3^/s (Figure 8).

**Figure 10:**
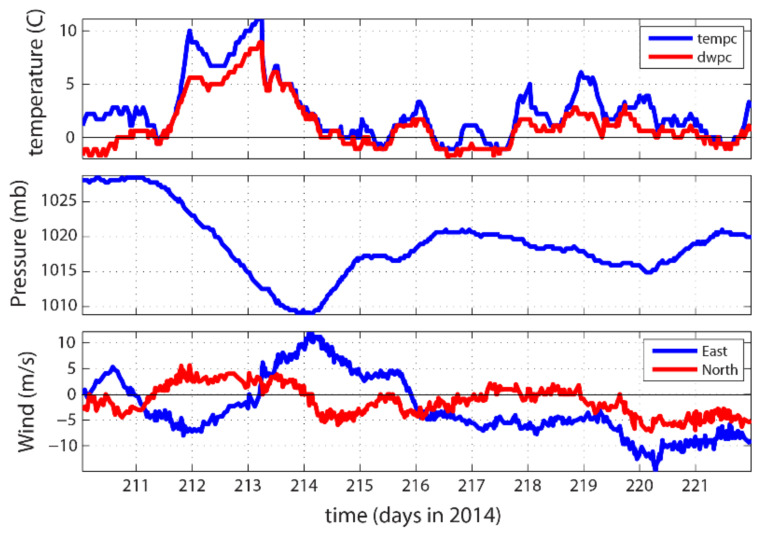
Weather time series data from the Barrow airport automated surface observation system (ASOS). The upper, middle, and lower panels are for the air temperature and dew point temperature, air pressure, and wind velocity components (east and north components); 2 August 2014 is day 214 starting from 1 January.

The weather data recorded by an automated surface observation system (ASOS) at the airport in Utqiaġvik showed this event. The local weather data showed that the event started with a warming of the ground level air from nearly 0 C to about 11 C in two days. The air temperature then dropped to below 0 C in another two days (upper panel of Figure 10). On 2 August (day 214 of 2014), the local sea level air pressure reached a minimum from about 1028 to 1009 mb (middle panel of Figure 10). Concurrently, wind speed increased and reached maximum almost at the same time when the air pressure reached its minimum. The wind direction was roughly northwest between Day 214 and Day 215 of 2014 (1 January was defined as Day 1). By analyzing the weather data for the whole year, the event of 2 August appeared to be the most intensive local event for the summer of 2014.

### 4.2. Comment about the Method

Ideally, if an ADCP can be used on a moving platform to acoustically measure the velocity profiles across a channel continuously, a reliable time series of transport can be obtained. This, however, is hardly realistic for most places because of the cost involved, not to mention the possibility in an environment with no infrastructure, and hazards associated with potential floating ice and freezing in the winter season. Alternatively, if an array of ADCPs can be deployed along the bottom, reliable measurements of transport over time is feasible. However, this is also problematic because of the cost involved to have multiple ADCPs and the risk of damage or loss in dynamic shallow water environments is very high in this region within the Arctic.

The advantage of the method presented in this paper is that it avoids conducting continuous measurements of the transport or using costly multiple ADCPs for bottom deployment. Instead, it establishes a correlation between the depth-averaged velocity from a bottom-mounted ADCP with a short-term vessel-based measurement of transport. This is particularly useful for applications in the Arctic because of the adverse environmental conditions there (low temperature, lack of facilities and even a standard boat launch).

Although the method has been shown to be valid and useful by our study, we would also like to add a few cautionary notes. The first is that the vessel-based surveys should be long enough to capture sufficient variability over time. In our study, the length was nearly 5 h and indicated a strong positive relationship. We expect that if the duration of the survey was longer (e.g., 24 h) it would be ideal as it would cover two tidal cycles. This method has been used in our previous studies in the Louisiana tidal channels [39] in which we had the luxury of having an entire diurnal tidal cycle (close to 25 h), which resulted in an even stronger relationship with R^2^ values ranging between 0.96 and 0.99. In the Arctic region, this is a novel experiment, and the results are encouraging. The method, however, has not been discussed in detail as in this paper. The work presented in [39] only presented the results with the context of examining weather-induced exchange flows through a tidal channel in southern Louisiana. This paper provides a detailed explanation for the first time with an actual challenging application in the Arctic.

### 4.3. On the Measurement Errors

*The use of bottom-tracking*. As described earlier, we used the bottom-tracking mode for the M9 ADCP. The original relative velocity between the moving vessel and the water is measured by the Doppler shift. This also considers the pitch and roll and orientation (with the onboard IMU and compass) and gives the three components of velocity profiles along the vertical. The velocity at each of the vertical positions has three components in the three-dimensional Cartesian xyz coordinate. However, that has not considered the velocity of the moving vessel. The velocity of the moving vessel is usually not calculated from the GPS because of relatively large errors from the raw GPS data—the ADCP needs to sample many times in a second (it varies with the M9, which, in our case, sampled 7-40 times per second, Table 1), and it is hard for a GPS to keep pace with the fast-updating requirements. Instead, the speed of the vessel is measured by “bottom-tracking”. This is a function of the ADCP using the same Doppler shift principle to measure the velocity of the sea bottom. This is generally more accurate than using the GPS for most applications unless a very high-resolution RTK GPS is used. After the bottom tracking velocity is obtained, it is subtracted from the xyz velocity to yield the Earth coordinate (ENU or East, North, Up) velocity components. The GPS unit used in this study is only for time stamping of the time series data and to link the bottom ADCP data with the vessel-based ADCP data. The position error is the general differential GPS error (3–6 m in position). However, since our footprint for the bottom ADCP is a rectangle of 74 × 47 m (Figure 5), the error in position is negligible.

**Figure 11:**
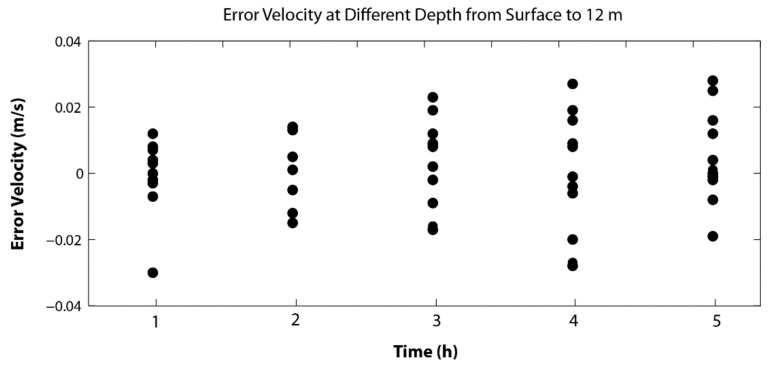
Velocity error from the bottom-mounted ADCP. The different dots at a given time are for the error velocity values at different depths from 1 to 12 m above the bottom (mab).

*The Errors for ADCP Data*. Both ADCPs provide error velocity estimates for each and every ensemble sample. For example, the error velocity from the bottom-mounted ADCP ranged from −0.03 to 0.03 m/s (Figure 11). The depth-averaged error velocity at each of the hours for the five hourly data are −0.0322, 0.0052, 0.0016, 0.0132, and 0.0152 m/s, respectively. The overall averaged error velocity is less than 0.01 m/s. Likewise, the error velocities of the M9 ADCP were also automatically computed by the instrument. The mean error velocity was computed to be less than 0.01 m/s with a standard deviation of ~0.07 m/s for the error. A greater range of error velocity compared to the bottom-mounted ADCP data is expected because of extra error introduced by the moving platform. This is consistent with previous studies. For example, the standard error for the velocity data from six full tidal-cycle surveys using a small research vessel in a tidal channel [40] was estimated to be between 0.09 and 0.17 m/s.

## 5. Concluding Remarks

The successful implementation of the proposed method in a coastal environment (e.g., tidal rivers, tidal inlets, estuaries, and straits) relies on several key factors:

(1) The time from all relevant equipment (bottom-mounted ADCP, vessel-based ADCP, and GPS) must be unified with the GPS time. UTC should be used to avoid confusion with the local time and/or daylight-saving time.
(2) The vessel-based ADCP should use the “bottom-tracking mode” unless the sea bottom is not solid (such as full of fluid mud). This in most cases will enhance the quality of the velocity data. In regions where water depth is too large and the sampling frequency of the ADCP is too high such that the ADCP could not sense the bottom, the “navigational mode” needs to be used, which in most cases might significantly increase the error of velocity measurements unless a high-resolution RTK GPS system is used. This is because of the random errors from the raw GPS, especially when high sampling rate is required for obtaining ADCP ensemble velocity values (e.g., 7-40 measurements are made to obtain a 1-s ensemble value for the M9 in our study, Table 1). In the case of using navigational mode, a temporal average of the ensemble velocity data can help in reducing the velocity error. Fortunately, this is unlikely in most coastal waters such as estuaries and lagoons because of their inherently shallow water. For example, the M9 ADCP can successfully use bottom-tracking mode in waters of 25 m. For a 600 KHz RDI ADCP, this depth can be increased to 60 m or more.
(3) The repeated measurements across the transect are very important to establish the statistical regression coefficients between the transport (from bottom-mounted ADCP) and velocity (from the vessel-based ADCP). In general, the more repetitions, the better.
(4) The temporal length of the measurements should be “long enough” to include certain variability of the flow velocity and total cross-sectional transport. In a tidal environment, this depends on the type of tides. The time should be long enough over which the flow velocity experiences sufficient variations for obtaining a reliable statistical regression. For a semi-diurnal tidal environment, the whole tidal cycle is about 12 h, and over 3–4 h, the flow can experience 1/4 to 1/3 of the one period for tidal currents, although measurements over a complete tidal cycle is preferred if possible [39].
(5) The cross-channel transect should pass the deployed ADCP: the closer the better. In choppy conditions, this might be difficult, but with numerous repetitions, enough valid samplings can be guaranteed.

In conclusion, using a combination of a longer-term bottom-mounted ADCP (the first ADCP), measuring the local velocity profiles in a deep channel of a tidal inlet and a shorter-term boat-based ADCP (the second ADCP) measuring the cross-channel transport continuously can allow us to establish a regression between the depth-averaged velocity from the first ADCP and the transport from the second ADCP. The regression coefficients can then be applied to the longer time series from the first ADCP and obtain the transport time series from the entire deployment. This appears to be an efficient and economical way to determine the total transport. This is useful particularly considering that during severe weather, a boat-based survey is usually not possible because of safety issues, unless a reliable automated unmanned platform is used, which can also be costly and has a high risk in a remote area such as the Arctic lagoons. This method can be used in many applications in the quantification of flux of water under tidal and weather forcing. This can be particularly useful in a system with multiple inlets so that coordinated observations can be made to quantify the fluxes through different inlets, which can help the understanding of the circulation dynamics and reliable quantification of the water exchange [22,38] of the system.

## Figures and Tables

**Table 1:** ADCP parameters.

ADCP	Bottom Mounted 1	Bottom Mounted 2	Vessel Based M9
Raw sampling interval (s)	80	6	variable (~1/7–1/40)
Ensemble interval	1 h	5 min	1 s
Vertical bin size (m)	1	0.25	variable

## Data Availability

Data for this paper are obtained by the authors in the Elson lagoon and can be found at https://oceandynamics.lsu.edu/Data.htm (accessed on 21 December 2021). Any questions should be directed to the authors.
